# A fatal presentation of DRESS syndrome with multiple visceral failure mimicking septic shock

**DOI:** 10.1016/j.amsu.2022.104110

**Published:** 2022-06-28

**Authors:** mohammed amine Lafkih, Hamza mimouni, mohammed Azizi, El Kaouini Abderrahim, mohammed Maarad, Bkiyar Houssam, Brahim Housni

**Affiliations:** a Faculty of Medicine and Pharmacy, Mohammed I^st^ University, Oujda, Morocco; b Department of Anesthesiology and Intensive Care Unit, Mohammed VI University Hospital Mohammed I University, Oujda, Morocco

**Keywords:** Dress syndrome, Allopurinol, Septic shock, Cutaneous adverse reaction to drug

## Abstract

**Introduction:**

The DRESS syndrome is a life-threatening multi-organ system reaction induced by drugs Characterized by a long latency between drug exposure and disease onset, allopurinol is the most incriminated drug.

**Case presentation:**

We report a case of 56-year-old patient with history of gout under allopurinol admitted in emergency for shock state associated erythematosquamous lesions reaching 65% of the body surface, a septic was suspected but the bacteriological investigations were negative and the patient had an isolated hyper eosinophilia so diagnosis of dress syndrome induced by allopurinol was retained The patient presented an acute renal failure that was treated successfully by renal replacement therapy, and corticosteroids.

**Discussion:**

DRESS syndrome has a mortality of 10–20%. Its clinical presentation is predominantly cutaneous, with or without visceral involvement. The cornerstone of the management of DRESS syndrome is the identification and discontinuation of the causative drug. Early diagnosis and screening for visceral involvement can reduce mortality.

**Conclusion:**

The DRESS syndrome is a severe adverse drug reaction and has high mortality rates Furthermore, judicious use of allopurinol may decrease its incidence.

## Highlights

• The DRESS syndrome is a life-threatening multi-organ system reaction induced by drugs Characterized by a long latency between drug exposure and disease onset.
• Allopurinol is the most incriminated drug.
• The DRESS syndrome is a severe adverse drug reaction and has high mortality rates Furthermore, judicious use of allopurinol may decrease its incidence.

## 1 Introduction

Severe cutaneous adverse reaction to drug (SCAR) are a severe adverse drug reactions, they contains generalized exanthematous pustulosis (AGEP) and Stevens-Johnson syndrome, toxic epidermal necrosis (TEN) and acute generalized exanthematous pustulosis (AGEP) The DRESS syndrome (Drug Reaction with Eosinophilia and Systemic Symptom) can cause a potentially life threatening organ dysfunction [1,2]. Many drugs can cause DRESS SYNDROME such us allopurinol which was taken by the patient in the case that we will present [2].

## 2 Case presentation

**Fig. 1:**
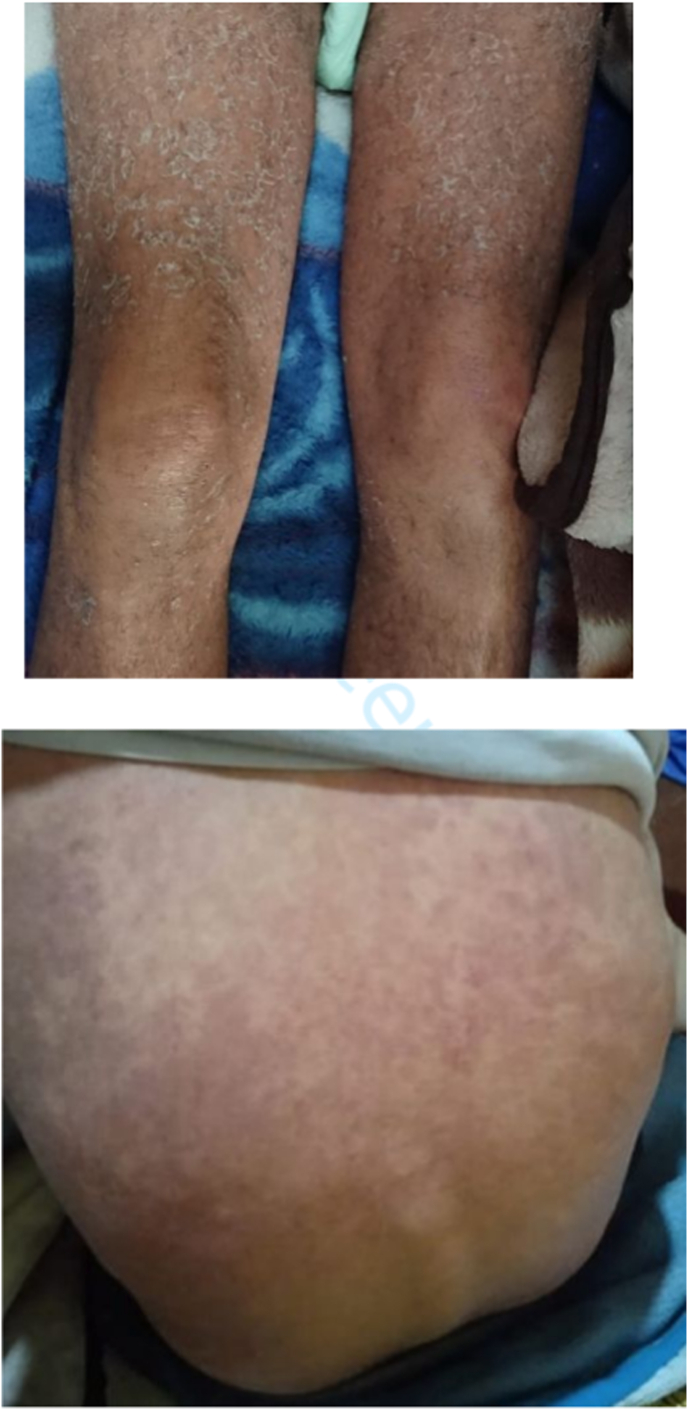
Erythematosquamous lesions reaching 65% of the body surface especially on the trunk, back, and face with negative candle sign and mucous membranes.

We report the case of a 56-year-old patient with history of gout under allopurinol since 10 years well controlled admitted to the emergency room for severe asthenia. The initial evaluation found a conscious patient GCS 15/15, hemodynamically unstable with Heart Rate: 130bpm Blood Pressure: 75/40 mmhg with signs of shock namely cold cyanotic extremities, respiratory polypneic patient at 26cpm SPO2 at room air at 90%, temperature at 39.1° capillary glycemia at 1.00 g/l. The patient is oligo-anuric. Clinical examination found erythematosquamous lesions reaching 65% of the body surface especially on the trunk, back, and face with negative candle sign and mucous membranes (Fig. 1), the rest of the cardiovascular, pulmonary examinations without partiality. The ECG showed sinus tachycardia, the chest X-ray was unremarkable and the arterial gasometer showed a metabolic acidosis with a hyperlactatemia of 4.8mmol/l. The patient was admitted to the intensive care unit after a filling test with isotonic saline at a dose of 30 ml/kg over 30 minutes without hemodynamic response. A right central jugular venous access was established, a right radial arterial line for invasive hemodynamic monitoring, the patient was hemodynamically stabilized on 0.4gamma/KG/min of norepinephrine. A transthoracic echocardiography was performed showing an uncomplicated inferior vena cava with preserved left ventricular systolic function without signs of right ventricular overload with a dry pericardium. At this stage, the diagnosis of septic shock was most likely due to the fever, shock, hyperlactatemia without signs of externalized hemorrhage or cardiac failure with a SOFA score calculated at 7. A biological check-up was carried out with negative Procalcitonin, CRP 90mg/l, Urea 2.05, Creatinine 35mg/l. The blood count showed an isolated hypereosinophilia of 4320 with the other blood lines being normal. The blood cultures taken during the febrile peaks twice came back negative and the rest of the infectious workup was negative. In view of the recent intake of allopurinol, the erythematosquamous skin lesions, hypereosinophilia, hemodynamic and visceral deficiency, and the negativity of all bacteriological explorations, the diagnosis of a DRESS syndrome with multivisceral involvement was made. Allopurinol was stopped and corticosteroid therapy was started. The evolution is marked by hemodynamic stability with weaning of vasoactive drugs with persistence of oligu-anuria with still high creatinine figures requiring continuous renal replacement therapy.

After 3 days of his admission, the patient recovered his renal function with a good clinico-biological evolution, and a complete weaning of the drugs, and then the patient is transferred in service of rheumatology for adjustment of his hypourecemiant treatment.

## 3 Discussion

The term DRESS (drug rush with eosinophilia and systemic symptoms) was initially introduced by bocquet [1], later we realized that it was a systemic reaction so it changed to DRESS (drug reaction with eosinophilia and systemic symptoms) [2], in fact the first symptoms were described in cases induced by anticonvulsant such us carbamazepine and dress syndrome was called at that time AHS (anticonvulsant hypersensivity syndrome) [3] The incidence of DRESS syndrome varies between 1/1000 and 1/10000 [4] with a mortality of 10–20% [5]. The clinical presentation of dress is predominantly cutaneous, with or without visceral involvement, with fever, adenopathy, elevated white blood count, and abnormal liver function. Fever is the most common symptom, often elevated between 38 and 40° [6]. A particular form has been reported: “typhus inversus” by Ben-Ari et al. where the temperature is high in the morning and low in the evening [7]. Skin manifestations are present in 73–100% of cases. [They are often urticarial or maculopapular lesions, but also vesicles, bullae, pustules, cheilitis, purpura and erythrodermia [8,9]. Facial edema, found in 74% of patients, is specific to the disease [10].

Liver involvement is the most common visceral involvement, accounting for 75–94% of cases, and Renal involvement is the second most common, occurring in 12–40% of cases [11]. It is often mild and non-sequential, but can in some cases cause renal failure through interstitial nephritis or acute tubular necrosis, [12]. Advanced age and underlying renal and cardiovascular disease increase the risk of renal damage [13]. The most common drug involved is allopurinol [14] as it was in our case.

Pulmonary involvement may manifest as interstitial lung disease, pleurisy, or ARDS. Cardiac involvement is manifested in two forms: hypersensitivity myocarditis and acute eosinophilic necrotizing myocarditis. Neurological involvement is caused by meningitis or encephalitis, and can be manifested by headache, convulsions, coma, motor disorders [15,]. The most commonly scoring system for diagnosing DRESS syndrome used is RegiSCAR criteria [16], there is also DIHS (drug induced hypersensitivity syndrome) criteria [17], and boquet criteria [18] The cornerstone of the management of DRESS syndrome is the identification and discontinuation of the causative drug. Early diagnosis and screening for visceral involvement can determine the therapeutic strategy and reduce mortality [19].

There are no randomized studies in the direction of DRESS treatment, recommendations are based on case reports and expert opinion. DRESS syndrome can be treated by anti-histamines, oral or systemic corticosteroids, IV immunoglobulins, plasmapheresis, valganciclovir, as well as maintenance of fluid and electrolyte balance and prevention of infections [19]. The healing process can take weeks after the drug was taken and it important that autoimmunity diseases should be searched [20].

The SCARE guidelines were used in the writing of this paper [21].

## 4 Conclusion

As DRESS is a rare life-threatening clinical entity, the main pillars of this disease's management are early diagnosis, cessation of the offending medication and supportive care. Judicious use of allopurinol may decrease the incidence and morbidity caused by this syndrome [20].

## Ethical approval

The ethical committee approval was not required give the article type (case report). However, the written consent to publish the clinical data of the patients was given and is available to check by the handling editor if needed.

## Source of funding

NONE.

## Author contribution

MOHAMMED AMINE LAFKIH: study concept or design, data collection, data analysis or interpretation, writing the paper. HAMZA MIMOUNI: Data collection, data analysis. AZIZI MOHAMMED: Data collection, data analysis. EL KAOUINI MOHAMEMD: Data collection, data analysis. MAARAD MOHAMMED: Data collection, data analysis. HOUSSAM BKIYAR: supervision and data validation. BRAHIM HOUSNI: supervision and data validation.

## Trail registry number

This is not an original research project involving human participants in an interventional or an observational study but a case report. This registration is was not required.

## Guarantor

LAFKIH MOHAMMED AMINE.

## Consent for publication

Written informed Consent was obtained from the patients for publication of this case report and accompanying images. A copy of the written consent is available for review by the Editor-in-Chief of this journal on request.

## Provenance and peer review

Not commissioned, externally peer reviewed.

## Declaration of competing interest

NONE.
